# Performance of newer myeloma staging systems in a contemporary, large patient cohort

**DOI:** 10.1038/s41408-024-01076-w

**Published:** 2024-06-11

**Authors:** Ghulam Rehman Mohyuddin, Samuel M. Rubinstein, Shaji Kumar, S. Vincent Rajkumar, Rafael Fonseca, Nadine H. Abdallah, Gregory S. Calip, Xiaoyan Wang, Christina M. Parrinello, Douglas Sborov

**Affiliations:** 1 Huntsman Cancer Institute, University of Utah, Salt lake, UT USA; 2 Lineberger Comprehensive Cancer Center, University of North Carolina, Chapel Hill, NC USA; 3 Division of Hematology, Mayo Clinic, Rochester, MN USA; 4 Division of Hematology, Mayo Clinic, Phoenix, AZ USA; 5 Titus Family Department of Clinical Pharmacy, University of Southern California, Los Angeles, CA USA; 6 Flatiron Health, New York, NY USA; 7 Pine Mountain Consulting, Larkspur, CA USA

**Keywords:** Myeloma, Epidemiology

**TO THE EDITOR:**

The most widely used staging system for patients with multiple myeloma (MM), the Revised International Staging System (R-ISS), is based on beta-2 microglobulin (B2M), albumin, lactate dehydrogenase (LDH), and the presence of high-risk cytogenetics [1]. However, it does not account for the additive risk imposed by multiple cytogenetic abnormalities or the presence of copy number gains at chromosome 1q21 (gain1q) [2]. The majority of patients (62%) are staged as R-ISS Stage II, with marked variation in prognosis depending on the co-occurrence of specific cytogenetic abnormalities [1, 3].

Two new staging systems, the Mayo Additive Staging System (MASS) [4], and the Second Revision of the International Staging System (R2-ISS), have been proposed [5], and incorporate additive risk resulting from the presence of multiple concurrent cytogenetic abnormalities [6], and adverse prognostic implications of gain1q. As both staging systems have not been validated concurrently in a large, contemporary US population, we externally validated MASS and R2-ISS and compared their performance to each other and to the R-ISS.

We conducted a retrospective cohort study using the nationwide Flatiron Health electronic health record (EHR)-derived database, which is a longitudinal database, comprising de-identified patient-level structured and unstructured data, curated via technology-enabled abstraction [7, 8]. During the study period, the de-identified data originated from up to 280 US cancer clinics (~800 sites of care) [7].This study was approved by the Institutional Review Board of the University of Utah.

We included patients who initiated first-line treatment for newly diagnosed MM between January 1st, 2016, and October 1st, 2022. Our primary outcome was overall survival (OS). Multivariable analysis was conducted using Cox models. All relevant tests were two-sided, and *p*-values < 0.05 were considered statistically significant. Analyses were conducted using R 4.1.3. A supplemental analysis was performed to evaluate the performance of these staging systems for time to next treatment (TTNT).

There were 497 patients with MM included. (Patient attrition is highlighted in Supplemental Table S1.) Patient characteristics are listed in Table S2. The majority of patients had received triplet therapy as induction (67.6%), with a minority receiving doublet (13.9%) and quadruplet regimens (10.7%). The majority of patients did not receive autologous stem cell transplantation during their disease course (66.4%) and were treated at community sites (82.3%). The median duration of follow-up of our cohort was 23.1 months (Q1: 10.9 months, Q3: 40.9 months).

The distribution of patients across R-ISS stages was as follows: 24%, 63%, and 13% for stage I, II and III respectively. The majority of the patients that had double-hit disease were classified as R-ISS Stage II (39/66, 59%). There was a statistically significant association between R-ISS stage and OS (log-rank p =<0.01) and the median OS was not reached (NR), 63 months, and 37 months for R-ISS stages I, II, and III respectively.

Patients were more evenly divided across MASS stages: 34%, 35% and 31% for MASS I, II, and III, respectively. All patients with double-hit disease were classified as MASS Stage III. There was a statistically significant association between MASS stage and OS (*p* ≤ 0.01), with median OS of 77, 61, and 45 months for MASS stage I, II, and III, respectively.

R2-ISS includes four risk categories (stages I-IV) and in our cohort, 20% were stage I (low), 25% were stage II (low-intermediate), 46% were stage III (intermediate), and 9% were stage IV (high). Most patients with double-hit disease were classified as Stage III (56%) or Stage IV (42%). There was a statistically significant association between R2-ISS and OS (*p* ≤ 0.01), though the survival curves for stage I and II were overlapping, as well as those for stage III and IV, suggesting that associations between stages I-II and OS were comparable, as were those between stages III-IV and OS. Median OS for R2-ISS stages I, II, III, and IV was NR, 69, 50 and 51 months, respectively (Fig. 1).

**Fig. 1:**
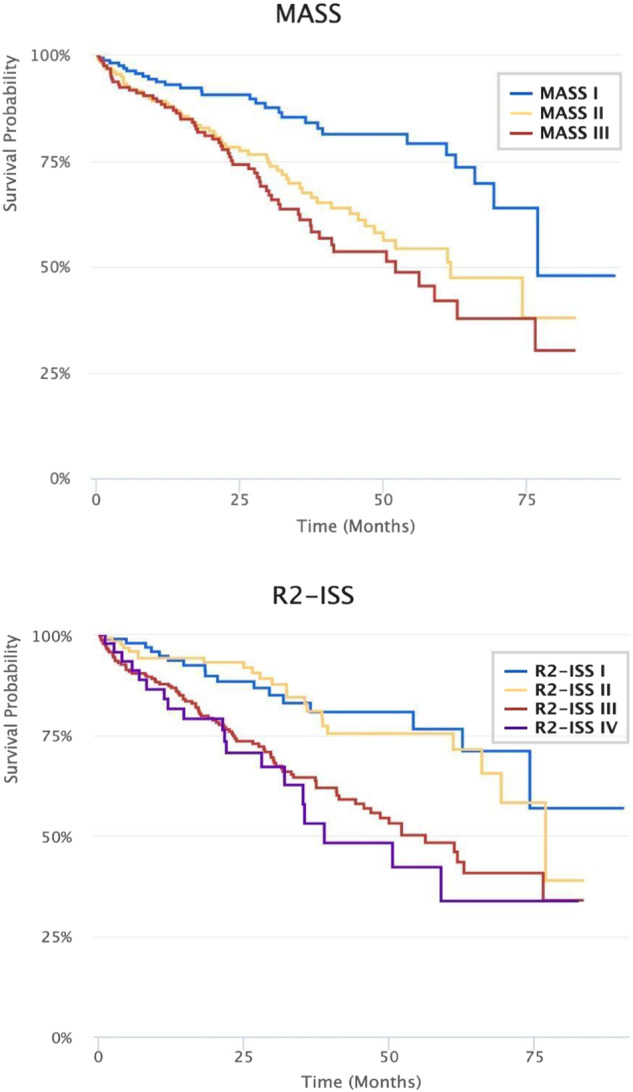
Kaplan-Meier product limit estimator survival functions for overall survival by MASS and R2-ISS stages.

As compared to R-ISS stage I, the adjusted hazard ratios (aHRs) for death were 1.8 (95%CI: 1.1, 2.9) and 2.9 (1.6, 5.3) for R-ISS stage II and stage III, respectively (Table S3). As compared to MASS I, the aHRs for death were 2.0 (95% CI 1.3-3.2) for MASS II and 2.7 (1.7–4.2) for MASS III (Table S4). The hazards of death were similar for R2-ISS stage I and stage II (aHR for II vs I: 1.2 [0.7, 2.3]). As compared to R2-ISS Stage I, the aHRs for death were similarly higher for stage III and stage IV (aHRs: 2.4 [1.4, 4.1] and 2.6 [1.3, 5.2], respectively) (Table S5). Discrimination and calibration were similar across all staging systems (*c* = 0.6, and Hosmer-Lemeshow *p* > 0.05 for all three staging systems) (Tables S3–5).

Migration between staging systems is described in Fig. 2, showing re-categorization of R-ISS Stage II into discrete MASS and R2-ISS stages. The supplement (Table S6-8) highlights performance of staging systems for TTNT, showing a shorter TTNT for Stage II and III (MASS) and Stage III and IV (R-2 ISS) compared to Stage 1- and a similar trend for R-ISS, although this did not achieve statistical significance. Table S9 lists treatment utilized across stages.

**Fig. 2:**
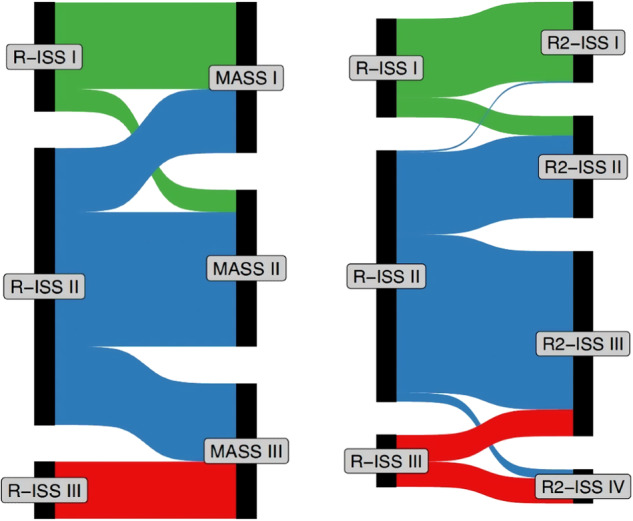
Sankey plot demonstrating redistribution of patients across different R-ISS stages (R-ISS to R2-ISS on left, R-ISS to MASS on right).

Overall survival was progressively worse as the number of high-risk cytogenetic abnormalities (HRCA) increased (0 vs 1 vs. 2+) as listed in Table S10, with a c-index of 0.55.

In this application of novel MM staging systems to a large, contemporary cohort of US patients, we demonstrate that they perform similar to R-ISS and retain prognostic capability for OS in MM, although their performance remains suboptimal with a c-index of ~0.6. Unlike R-ISS, for which most patients fall into stage II, MASS, and R2-ISS result in a wide distribution of patients across different stages. MASS demonstrated a greater separation for risk of mortality across stages, whereas R2-ISS stage III and stage IV were largely overlapping regarding risk of mortality.

A key limitation of R-ISS is that it categorizes the majority of patients into R-ISS stage II [1].Although it is well known that R-ISS stage III confers a poor prognosis, there is significant heterogeneity within R-ISS stage II [3]. Patients with zero, one, or multiple high-risk cytogenetic abnormalities all may be categorized within R-ISS stage II [3]. Although our analysis demonstrates that the prognostic value of the novel staging systems is similar to R-ISS, a key advantage is that these newer staging systems reclassify patients from R-ISS stage II into more refined risk-stratified subsets. Patients with double-hit disease are invariably staged as high-risk (Stage III or IV) with newer risk stratification models.

Our cohort was different from the derivative cohort of each of the staging systems, with a median age of 70, as opposed to an age of 60 in the R2-ISS derivation cohort [5]; and mostly at community sites, as opposed to the MASS staging system, which was developed using data from Mayo Clinic [4]. As such, our cohort is reflective of a heterogenous contemporary US cohort of patients largely receiving modern triplet therapy and externally validates the use of these staging systems in practice where modern therapy incorporating triplet therapy is used.

Our work represents the first comparison of these newer staging systems to each other, and the first to externally validate both of them simultaneously in a large multi-center contemporary United States cohort treated with modern triplet therapy. Previous work has evaluated these staging systems in small single-center analyses, such as two single-center reports from China evaluating the performance of MASS in 94 [9] and 307 patients [10], respectively. Similar single-center studies have also evaluated the performance of R2-ISS in cohorts from China and Japan [11, 12]. An analysis in the United States has validated R2-ISS, but this was in a transplanted cohort, different from our analysis, which was predominantly in a non-transplant cohort [13].

Our dataset has various limitations. The median duration of follow-up was 23.1 months, which is not enough time to accrue many events, given the advances in therapy and improved outcomes in this patient population [14]. We therefore evaluated TTNT in addition to OS. Our dataset also does not account for copy numbers of Gain1q, and we cannot differentiate between Amp1q versus Gain1q, which may have different prognostic implications, although data is conflicting in this regard [15]. A limitation of all staging systems across MM is that rather than conventional staging systems in solid tumors that reflect tumor burden and spread, these systems serve instead as risk-stratification models. They group together heterogenous variables of biological aggressiveness, patient characteristics, and tumor burden. Although they are very useful for prognostication purposes, given the heterogeneity of patients within each stage, they are less useful in individualizing therapy for different subsets of patients. Furthermore, our analysis is only applicable to patients who received treatment, as we excluded patients who did not receive any therapy.

In summary, this study demonstrates that both R2-ISS and MASS perform similarly in a contemporary cohort of US patients with MM and re-classify patients with R-ISS stage II MM into more refined prognostic subsets. As all models (including stratification per number of HRCA) perform with c-statistic close to 0.6, their performance remains suboptimal. Further validation of these staging systems in datasets with longer follow-up and more use of quadruplet therapy will be needed.

### Supplementary information

Supplemental Material

## Data Availability

Data can be shared upon a reasonable request to the corresponding author.
