# Supplementary material for: Performance of newer myeloma staging systems in a contemporary, large patient cohort
Source: Blood Cancer J. 2024 Jun 11;14(1):95. doi: 10.1038/s41408-024-01076-w (PMC11166956; doi:10.1038/s41408-024-01076-w)
Supplement: Supplementary file 1 — Supplemental Material [file 41408_2024_1076_MOESM1_ESM.docx]

**SUPPLEMENT**

**Table S1.** Attrition table

| **n** | **Inclusion Criterion** |
| --- | --- |
| 16,522 | In the Flatiron Health EHR-derived MM database |
| 7,895 | Has a first-line start date between January 1, 2016 and October 1, 2022 |
| 4,967 | Has complete information for ISS staging |
| 874 | Has FISH results available within the relevant time window |
| 497 | Has LDH results available within the relvant time window |

| **Table S2.** Baseline descriptive and clinical characteristics of patients with NDMM at initiation of first-line therapy | | |
| --- | --- | --- |
|  | **All NDMM Patients (N=497)** | |
|  | n | (%) |
| **Age, years** |  |  |
| Median (IQR) | 70 | (62-76) |
| **Sex** |  |  |
| Female | 234 | (47.1) |
| Male | 263 | (52.9) |
| **Race/ethnicity** |  |  |
| Hispanic | 23 | (4.6) |
| Non-Hispanic Asian | 5 | (1.0) |
| Non-Hispanic Black | 84 | (16.9) |
| Non-Hispanic Other | 23 | (4.6) |
| Non-Hispanic White | 326 | (65.6) |
| Unknown | 36 | (7.2) |
| **Practice type** |  |  |
| Community | 409 | (82.3) |
| Academic | 88 | (17.7) |
| **ECOG PS^1^** |  |  |
| 0 | 157 | (31.6) |
| 1 | 177 | (35.6) |
| 2-4 | 84 | (16.9) |
| Unknown | 79 | (15.9) |
| **First-line treatment type^2^** |  |  |
| Doublet | 69 | (13.9) |
| Triplet | 336 | (67.6) |
| Quad | 53 | (10.7) |
| Other | 39 | (7.8) |
| **ASCT** |  |  |
| Up to 1-year post-initiation | 150 | (30.2) |
| >1-year post-initiation | 17 | (3.4) |
| Not transplanted | 330 | (66.4) |
| **High-risk cytogenetics** |  |  |
| Translocation (4;14) | 47 | (9.5) |
| Translocation (14;16) | 28 | (5.6) |
| Translocation (14;20) | 9 | (1.8) |
| 1q gain / amplification | 167 | (33.6) |
| Deletion 17p | 46 | (9.3) |
| **Double-hit status for high-risk cytogenetics** |  |  |
| Double-hit | 66 | (13.3) |
| No double-hit | 431 | (86.7) |
| *Abbreviations:* ASCT, autologous stem cell transplantation; ECOG PS, Eastern Cooperative Oncology Group Performance Status; IQR, interquartile range; NDMM, newly diagnosed multiple myeloma  1. ECOG PS was identified as the value closest to the index date (within 30 days before and up to 7 days after the index date)  2. Patients receiving either monotherapy or a clinical study drug were categorized as “Other” | | |

| **Table S3.** Prognostic performance of R-ISS risk-stratified groups overall and by age, transplantation status and year of diagnosis subgroups | | | | | | | | | |
| --- | --- | --- | --- | --- | --- | --- | --- | --- | --- |
| **R-ISS risk group** | **Number of patients** | **Median OS (months)** | **Crude HR** | **(95% CI)** | **P** | **Adjusted HR*** | **(95% CI)** | **P** | **Harrell’s C-Index** |
| *All patients* |  |  |  |  |  |  |  |  |  |
| Stage I | 122 | NR | Reference |  |  | Reference |  |  |  |
| Stage II | 311 | 62.9 | 1.84 | (1.15, 2.97) | 0.01 | 1.80 | (1.11, 2.91) | 0.02 | 0.58 |
| Stage III | 64 | 37.5 | 3.03 | (1.70, 5.40) | <0.01 | 2.94 | (1.64, 5.26) | <0.01 |  |
| *Ages <65 years* |  |  |  |  |  |  |  |  |  |
| Stage I | 43 | 74.2 | Reference |  |  | Reference |  |  |  |
| Stage II | 91 | NR | 1.18 | (0.49, 2.85) | 0.72 | 1.00 | (0.39, 2.59) | 1.00 | 0.53 |
| Stage III | 20 | 76.5 | 1.37 | (0.40, 4.72) | 0.62 | 1.07 | (0.28, 4.06) | 0.92 |  |
| *Ages 65-74 years* |  |  |  |  |  |  |  |  |  |
| Stage I | 50 | NR | Reference |  |  | Reference |  |  |  |
| Stage II | 120 | 69.3 | 1.89 | (0.84, 4.30) | 0.13 | 1.93 | (0.83, 4.48) | 0.13 | 0.58 |
| Stage III | 22 | 32.0 | 3.67 | (1.36, 9.89) | 0.01 | 2.74 | (0.98, 7.69) | 0.06 |  |
| *Ages 75+ years* |  |  |  |  |  |  |  |  |  |
| Stage I | 29 | NR | Reference |  |  | Reference |  |  |  |
| Stage II | 100 | 44.2 | 2.12 | (0.95, 4.72) | 0.07 | 2.26 | (0.99, 5.15) | 0.05 | 0.58 |
| Stage III | 22 | 21.4 | 3.60 | (1.43, 9.06) | 0.01 | 3.49 | (1.30, 9.36) | 0.01 |  |
| *Received transplant* |  |  |  |  |  |  |  |  |  |
| Stage I | 41 | NR | Reference |  |  | Reference |  |  |  |
| Stage II | 93 | NR | 2.37 | (0.68, 8.27) | 0.18 | 1.99 | (0.54, 7.34) | 0.30 | 0.59 |
| Stage III | 16 | 76.5 | 3.60 | (0.78, 16.5) | 0.10 | 2.45 | (0.46, 13.00) | 0.29 |  |
| *Never received transplant* |  |  |  |  |  |  |  |  |  |
| Stage I | 81 | 74.2 | Reference |  |  | Reference |  |  |  |
| Stage II | 218 | 48.5 | 1.70 | (1.02, 2.85) | 0.04 | 1.67 | (0.99, 2.83) | 0.05 | 0.57 |
| Stage III | 48 | 31.7 | 2.61 | (1.40, 4.88) | <0.01 | 2.79 | (1.48, 5.25) | <0.01 |  |
| *Diagnosed in 2016-2019* |  |  |  |  |  |  |  |  |  |
| Stage I | 58 | NR | Reference |  |  | Reference |  |  | 0.59 |
| Stage II | 146 | 62.9 | 2.13 | (1.17, 3.87) | 0.01 | 2.04 | (1.11, 3.74) | 0.02 |  |
| Stage III | 30 | 38.8 | 3.47 | (1.67, 7.23) | <0.01 | 3.14 | (1.49, 6.62) | <0.01 |  |
| Diagnosed in 2020-2022 |  |  |  |  |  |  |  |  |  |
| Stage I | 64 | NR | Reference |  |  | Reference |  |  | 0.57 |
| Stage II | 165 | NR | 1.39 | (0.63, 3.06) | 0.41 | 1.47 | (0.66, 3.28) | 0.34 |  |
| Stage III | 34 | 32.0 | 2.29 | (0.90, 5.82) | 0.08 | 2.48 | (0.96, 6.38) | 0.06 |  |
| *Double-hit* |  |  |  |  |  |  |  |  |  |
| Stage I | 0 | -- | -- | -- | -- | -- | -- | -- | 0.54 |
| Stage II | 39 | 50.6 | Reference |  |  | Reference |  |  |  |
| Stage III | 27 | 38.8 | 1.31 | (0.62, 2.78) | 0.48 | 1.05 | (0.48, 2.30) | 0.91 |  |
| *No double-hit* |  |  |  |  |  |  |  |  |  |
| Stage I | 122 | NR | Reference |  |  | Reference |  |  | 0.58 |
| Stage II | 272 | 65.9 | 1.76 | (1.08, 2.86) | 0.02 | 1.68 | (1.03, 2.75) | 0.04 |  |
| Stage III | 37 | 37.5 | 2.94 | (1.49, 5.80) | <0.01 | 3.07 | (1.54, 6.12) | <0.01 |  |
| *Abbreviations:* CI, confidence interval; HR, hazard ratio; NR, not reached; OS, overall survival; R-ISS, Revised International Staging System  *The multivariable model that included all patients was adjusted for sex, race/ethnicity, practice type and diagnosis year; and was stratified by age group. The models by age subgroup do not include adjustment or stratification by age. The models by diagnosis year do not include adjustment for diagnosis year. | | | | | | | | | |

| **Table S4.** Prognostic performance of MASS risk-stratified groups overall and by age, transplantation status and year of diagnosis subgroups | | | | | | | | | |
| --- | --- | --- | --- | --- | --- | --- | --- | --- | --- |
| **MASS risk group** | **Number of patients** | **Median OS (months)** | **Crude HR** | **(95% CI)** | **P** | **Adjusted HR*** | **(95% CI)** | **P** | **Harrell’s C-Index** |
| *All patients* |  |  |  |  |  |  |  |  |  |
| Stage I | 169 | 76.9 | Reference |  |  | Reference |  |  |  |
| Stage II | 176 | 61.2 | 2.14 | (1.36, 3.38) | <0.01 | 2.00 | (1.26, 3.17) | <0.01 | 0.60 |
| Stage III | 152 | 45.0 | 2.72 | (1.73, 4.27) | <0.01 | 2.66 | (1.69, 4.19) | <0.01 |  |
| *Ages <65 years* |  |  |  |  |  |  |  |  |  |
| Stage I | 60 | NR | Reference |  |  | Reference |  |  |  |
| Stage II | 47 | 74.2 | 1.72 | (0.68, 4.36) | 0.25 | 1.62 | (0.60, 4.37) | 0.34 | 0.58 |
| Stage III | 47 | 76.5 | 1.74 | (0.70, 4.34) | 0.24 | 1.51 | (0.54, 4.20) | 0.43 |  |
| *Ages 65-74 years* |  |  |  |  |  |  |  |  |  |
| Stage I | 62 | 69.3 | Reference |  |  | Reference |  |  |  |
| Stage II | 74 | NR | 2.34 | (1.02, 5.36) | 0.04 | 2.05 | (0.87, 4.87) | 0.10 | 0.62 |
| Stage III | 56 | 45.0 | 3.81 | (1.68, 8.65) | <0.01 | 3.23 | (1.40, 7.44) | 0.01 |  |
| *Ages 75+ years* |  |  |  |  |  |  |  |  |  |
| Stage I | 47 | 76.9 | Reference |  |  | Reference |  |  |  |
| Stage II | 55 | 33.5 | 2.06 | (1.02, 4.15) | 0.04 | 2.41 | (1.17, 4.97) | 0.02 | 0.57 |
| Stage III | 49 | 27.6 | 2.37 | (1.19, 4.73) | 0.01 | 2.26 | (1.09, 4.65) | 0.03 |  |
| *Received transplant* |  |  |  |  |  |  |  |  |  |
| Stage I | 55 | NR | Reference |  |  | Reference |  |  |  |
| Stage II | 47 | NR | 0.86 | (0.22, 3.35) | 0.83 | 0.85 | (0.21, 3.49) | 0.82 | 0.68 |
| Stage III | 48 | 76.5 | 2.98 | (1.16, 7.66) | 0.02 | 3.14 | (1.10, 8.98) | 0.03 |  |
| *Never received transplant* |  |  |  |  |  |  |  |  |  |
| Stage I | 114 | 76.9 | Reference |  |  | Reference |  |  |  |
| Stage II | 129 | 38.4 | 2.20 | (1.33, 3.64) | <0.01 | 1.99 | (1.19, 3.33) | <0.01 | 0.59 |
| Stage III | 104 | 37.5 | 2.61 | (1.56, 4.35) | <0.01 | 2.54 | (1.51, 4.26) | <0.01 |  |
| *Diagnosed in 2016-2019* |  |  |  |  |  |  |  |  |  |
| Stage I | 85 | 76.9 | Reference |  |  | Reference |  |  | 0.63 |
| Stage II | 76 | 61.2 | 2.31 | (1.33, 4.01) | <0.01 | 2.22 | (1.26, 3.93) | <0.01 |  |
| Stage III | 73 | 45.0 | 3.09 | (1.79, 5.33) | <0.01 | 2.97 | (1.71, 5.16) | <0.01 |  |
| *Diagnosed in 2020-2022* |  |  |  |  |  |  |  |  |  |
| Stage I | 84 | NR | Reference |  |  | Reference |  |  | 0.57 |
| Stage II | 100 | NR | 1.76 | (0.79, 3.91) | 0.17 | 1.83 | (0.81, 4.15) | 0.15 |  |
| Stage III | 79 | NR | 2.00 | (0.90, 4.46) | 0.09 | 2.18 | (0.96, 4.96) | 0.06 |  |
| *Double-hit* |  |  |  |  |  |  |  |  |  |
| Stage I | 0 | -- | -- | -- | -- | -- | -- | -- | -- |
| Stage II | 0 | -- | -- | -- | -- | -- | -- | -- |  |
| Stage III | 66 | 41.4 | -- | -- | -- | -- | -- | -- |  |
| *No double-hit* |  |  |  |  |  |  |  |  |  |
| Stage I | 169 | 76.9 | Reference |  |  | Reference |  |  | 0.60 |
| Stage II | 176 | 61.2 | 2.11 | (1.34, 3.33) | <0.01 | 1.93 | (1.22, 3.08) | <0.01 |  |
| Stage III | 86 | 56.2 | 2.62 | (1.55, 4.42) | <0.01 | 2.63 | (1.54, 4.47) | <0.01 |  |
| *Abbreviations:* CI, confidence interval; HR, hazard ratio; MASS, Mayo Additive Staging System; NR, not reached; OS, overall survival  *The multivariable model that included all patients was adjusted for sex, race/ethnicity, practice type and diagnosis year; and was stratified by age group. The models by age subgroup do not include adjustment or stratification by age. The models by diagnosis year do not include adjustment for diagnosis year. | | | | | | | | | |

| **Table S5.** Prognostic performance of R2-ISS risk stratified groups overall and by age, transplantation status and year of diagnosis subgroups | | | | | | | | | |
| --- | --- | --- | --- | --- | --- | --- | --- | --- | --- |
| **R2-ISS risk group** | **Number of patients** | **Median OS (months)** | **Crude HR** | **(95% CI)** | **P** | **Adjusted HR*** | **(95% CI)** | **P** | **Harrell’s C-Index** |
| *All patients* |  |  |  |  |  |  |  |  |  |
| Stage I | 100 | NR | Reference |  |  | Reference |  |  |  |
| Stage II | 126 | 69.3 | 1.19 | (0.64, 2.21) | 0.59 | 1.22 | (0.65, 2.29) | 0.53 | 0.61 |
| Stage III | 229 | 50.0 | 2.52 | (1.49, 4.26) | <0.01 | 2.40 | (1.41, 4.06) | <0.01 |  |
| Stage IV | 42 | 50.6 | 2.78 | (1.40, 5.50) | <0.01 | 2.62 | (1.31, 5.24) | 0.01 |  |
| *Ages <65 years* |  |  |  |  |  |  |  |  |  |
| Stage I | 36 | 74.2 | Reference |  |  | Reference |  |  |  |
| Stage II | 44 | NR | 0.91 | (0.29, 2.83) | 0.86 | 0.83 | (0.26, 2.67) | 0.75 | 0.57 |
| Stage III | 65 | 76.5 | 1.44 | (0.55, 3.77) | 0.45 | 1.22 | (0.43, 3.48) | 0.71 |  |
| Stage IV | 9 | 50.6 | 2.04 | (0.41, 10.20) | 0.39 | 1.29 | (0.24, 7.11) | 0.77 |  |
| *Ages 65-74 years* |  |  |  |  |  |  |  |  |  |
| Stage I | 40 | NR | Reference |  |  | Reference |  |  |  |
| Stage II | 50 | 69.3 | 1.47 | (0.50, 4.30) | 0.49 | 1.64 | (0.55, 4.91) | 0.38 | 0.62 |
| Stage III | 84 | 56.2 | 2.67 | (1.03, 6.97) | 0.04 | 2.48 | (0.93, 6.58) | 0.07 |  |
| Stage IV | 18 | 32.0 | 4.31 | (1.36, 13.6) | 0.01 | 4.16 | (1.30, 13.30) | 0.02 |  |
| *Ages 75+ years* |  |  |  |  |  |  |  |  |  |
| Stage I | 24 | NR | Reference |  |  | Reference |  |  |  |
| Stage II | 32 | 61.0 | 1.26 | (0.44, 3.64) | 0.67 | 1.14 | (0.39, 3.35) | 0.81 | 0.59 |
| Stage III | 80 | 29.7 | 2.83 | (1.19, 6.72) | 0.02 | 2.97 | (1.22, 7.23) | 0.02 |  |
| Stage IV | 15 | 35.5 | 2.01 | (0.67, 5.99) | 0.21 | 1.89 | (0.60, 5.96) | 0.28 |  |
| *Received transplant* |  |  |  |  |  |  |  |  |  |
| Stage I | 32 | NR | Reference |  |  | Reference |  |  |  |
| Stage II | 43 | 69.3 | 1.16 | (0.26, 5.27) | 0.85 | 1.18 | (0.25, 5.49) | 0.84 | 0.66 |
| Stage III | 61 | 76.5 | 2.32 | (0.64, 8.35) | 0.20 | 1.97 | (0.49, 8.00) | 0.34 |  |
| Stage IV | 14 | 50.6 | 6.68 | (1.44, 30.9) | 0.02 | 5.84 | (1.19, 28.80) | 0.03 |  |
| *Never received transplant* |  |  |  |  |  |  |  |  |  |
| Stage I | 68 | 74.2 | Reference |  |  | Reference |  |  |  |
| Stage II | 83 | 76.9 | 1.25 | (0.63, 2.48) | 0.52 | 1.25 | (0.62, 2.49) | 0.54 | 0.60 |
| Stage III | 168 | 37.5 | 2.43 | (1.36, 4.31) | <0.01 | 2.29 | (1.27, 4.10) | 0.01 |  |
| Stage IV | 28 | 35.5 | 2.36 | (1.09, 5.10) | 0.03 | 2.27 | (1.03, 5.00) | 0.04 |  |
| *Diagnosed in 2016-2019* |  |  |  |  |  |  |  |  |  |
| Stage I | 51 | NR | Reference |  |  | Reference |  |  | 0.62 |
| Stage II | 52 | 69.3 | 1.21 | (0.57, 2.55) | 0.63 | 1.18 | (0.55, 2.55) | 0.67 |  |
| Stage III | 110 | 48.5 | 2.65 | (1.42, 4.96) | <0.01 | 2.53 | (1.35, 4.75) | <0.01 |  |
| Stage IV | 21 | 50.6 | 2.61 | (1.13, 6.05) | 0.03 | 2.27 | (0.96, 5.34) | 0.06 |  |
| *Diagnosed in 2020-2022* |  |  |  |  |  |  |  |  |  |
| Stage I | 49 | NR | Reference |  |  | Reference |  |  | 0.61 |
| Stage II | 74 | NR | 1.13 | (0.37, 3.45) | 0.83 | 1.20 | (0.38, 3.74) | 0.76 |  |
| Stage III | 119 | NR | 2.23 | (0.86, 5.81) | 0.10 | 2.40 | (0.90, 6.40) | 0.08 |  |
| Stage IV | 21 | 32.0 | 2.98 | (0.91, 9.78) | 0.07 | 3.36 | (1.00, 11.30) | 0.05 |  |
| *Double-hit* |  |  |  |  |  |  |  |  |  |
| Stage I | 0 | -- | -- |  |  | -- |  |  | 0.54 |
| Stage II | 1 | NR | -- |  |  | -- |  |  |  |
| Stage III | 37 | 41.4 | Reference |  |  | Reference |  |  |  |
| Stage IV | 28 | 38.8 | 1.40 | (0.65, 3.02) | 0.38 | 0.92 | (0.39, 2.19) | 0.86 |  |
| *No double-hit* |  |  |  |  |  |  |  |  |  |
| Stage I | 100 | NR | Reference |  |  | Reference |  |  | 0.62 |
| Stage II | 125 | 69.3 | 1.19 | (0.64, 2.22) | 0.58 | 1.21 | (0.65, 2.28) | 0.55 |  |
| Stage III | 192 | 50.0 | 2.51 | (1.47, 4.30) | <0.001 | 2.30 | (1.34, 3.95) | <0.01 |  |
| Stage IV | 14 | NR | 1.77 | (0.59, 5.28) | 0.30 | 1.80 | (0.59, 5.47) | 0.30 |  |
| *Abbreviations:* CI, confidence interval; HR, hazard ratio; NR, not reached; OS, overall survival; R2-ISS, Second Revision of the International Staging System  *The multivariable model that included all patients was adjusted for sex, race/ethnicity, practice type and diagnosis year; and was stratified by age group. The models by age subgroup do not include adjustment or stratification by age. The models by diagnosis year do not include adjustment for diagnosis year. | | | | | | | | | |

| **Table S6.** Time to treatment in R-ISS risk-stratified groups overall and by age, transplantation status, year of diagnosis and double-hit status subgroups | | | | | | | | | |
| --- | --- | --- | --- | --- | --- | --- | --- | --- | --- |
| **R-ISS risk group** | **Number of patients** | **Median TTNT (months)** | **Crude HR** | **(95% CI)** | **P** | **Adjusted HR*** | **(95% CI)** | **P** | **Harrell’s C-Index** |
| *All patients* |  |  |  |  |  |  |  |  |  |
| Stage I | 122 | 18.2 | Reference |  |  | Reference |  |  |  |
| Stage II | 311 | 13.4 | 1.20 | (0.92, 1.57) | 0.18 | 1.28 | (0.98, 1.68) | 0.07 | 0.52 |
| Stage III | 64 | 14.6 | 1.38 | (0.95, 2.00) | 0.09 | 1.46 | (0.99, 2.14) | 0.05 |  |
| *Ages <65 years* |  |  |  |  |  |  |  |  |  |
| Stage I | 43 | 11.4 | Reference |  |  | Reference |  |  |  |
| Stage II | 91 | 9.7 | 1.01 | (0.65, 1.57) | 0.96 | 1.12 | (0.70, 1.78) | 0.64 | 0.51 |
| Stage III | 20 | 12.9 | 1.03 | (0.53, 2.02) | 0.92 | 1.18 | (0.58, 2.41) | 0.65 |  |
| *Ages 65-74 years* |  |  |  |  |  |  |  |  |  |
| Stage I | 50 | 24.0 | Reference |  |  | Reference |  |  |  |
| Stage II | 120 | 15.7 | 1.52 | (0.94, 2.47) | 0.09 | 1.68 | (1.02, 2.78) | 0.04 | 0.56 |
| Stage III | 22 | 9.8 | 2.44 | (1.27, 4.70) | <0.01 | 2.35 | (1.19, 4.63) | 0.01 |  |
| *Ages 75+ years* |  |  |  |  |  |  |  |  |  |
| Stage I | 29 | 14.0 | Reference |  |  | Reference |  |  |  |
| Stage II | 100 | 13.4 | 1.10 | (0.69, 1.77) | 0.69 | 0.94 | (0.57, 1.57) | 0.82 | 0.51 |
| Stage III | 22 | 16.8 | 0.96 | (0.51, 1.83) | 0.91 | 0.79 | (0.39, 1.59) | 0.51 |  |
| *Received transplant* |  |  |  |  |  |  |  |  |  |
| Stage I | 41 | 25.2 | Reference |  |  | Reference |  |  |  |
| Stage II | 93 | 11.7 | 1.18 | (0.73, 1.92) | 0.50 | 1.36 | (0.81, 2.30) | 0.25 | 0.53 |
| Stage III | 16 | 9.8 | 1.85 | (0.92, 3.73) | 0.09 | 2.08 | (0.97, 4.50) | 0.06 |  |
| *Never received transplant* |  |  |  |  |  |  |  |  |  |
| Stage I | 81 | 18.2 | Reference |  |  | Reference |  |  |  |
| Stage II | 218 | 14.0 | 1.18 | (0.86, 1.63) | 0.30 | 1.22 | (0.88, 1.69) | 0.24 | 0.52 |
| Stage III | 48 | 14.7 | 1.18 | (0.76, 1.84) | 0.47 | 1.23 | (0.78, 1.95) | 0.37 |  |
| *Diagnosed in 2016-2019* |  |  |  |  |  |  |  |  |  |
| Stage I | 58 | 19.6 | Reference |  |  | Reference |  |  | 0.53 |
| Stage II | 146 | 15.3 | 1.14 | (0.80, 1.62) | 0.48 | 1.25 | (0.87, 1.79) | 0.23 |  |
| Stage III | 30 | 12.4 | 1.69 | (1.03, 2.79) | 0.04 | 1.87 | (1.11, 3.16) | 0.02 |  |
| Diagnosed in 2020-2022 |  |  |  |  |  |  |  |  |  |
| Stage I | 64 | 18.1 | Reference |  |  | Reference |  |  | 0.53 |
| Stage II | 165 | 11.7 | 1.28 | (0.86, 1.91) | 0.22 | 1.34 | (0.89, 2.02) | 0.16 |  |
| Stage III | 34 | 21.4 | 1.11 | (0.63, 1.95) | 0.73 | 1.11 | (0.63, 1.98) | 0.72 |  |
| *Double-hit* |  |  |  |  |  |  |  |  |  |
| Stage I | 0 | -- | -- | -- | -- | -- | -- | -- | 0.50 |
| Stage II | 39 | 13.1 | Reference |  |  | Reference |  |  |  |
| Stage III | 27 | 14.7 | 1.09 | (0.60, 2.00) | 0.78 | 1.14 | (0.58, 2.25) | 0.70 |  |
| *No double-hit* |  |  |  |  |  |  |  |  |  |
| Stage I | 122 | 18.2 | Reference |  |  | Reference |  |  | 0.53 |
| Stage II | 272 | 13.6 | 1.20 | (0.92, 1.58) | 0.18 | 1.26 | (0.95, 1.66) | 0.11 |  |
| Stage III | 37 | 14.3 | 1.48 | (0.95, 2.31) | 0.08 | 1.53 | (0.97, 2.41) | 0.07 |  |
| *Abbreviations:* CI, confidence interval; HR, hazard ratio; NR, not reached; R-ISS, Revised International Staging System; TTNT, time to next treatment  *The multivariable model that included all patients was adjusted for age, sex, practice type and diagnosis year; and race/ethnicity was included as a stratification factor. The models by age subgroup do not include adjustment for age. The models by diagnosis year do not include adjustment for diagnosis year. | | | | | | | | | |

| **Table S7.** Prognostic performance of MASS risk-stratified groups overall and by age, transplantation status, year of diagnosis and double-hit status subgroups | | | | | | | | | |
| --- | --- | --- | --- | --- | --- | --- | --- | --- | --- |
| **MASS risk group** | **Number of patients** | **Median TTNT (months)** | **Crude HR** | **(95% CI)** | **P** | **Adjusted HR*** | **(95% CI)** | **P** | **Harrell’s C-Index** |
| *All patients* |  |  |  |  |  |  |  |  |  |
| Stage I | 169 | 20.2 | Reference |  |  | Reference |  |  |  |
| Stage II | 176 | 11.7 | 1.53 | (1.17, 2.00) | <0.01 | 1.52 | (1.15, 2.01) | <0.01 | 0.54 |
| Stage III | 152 | 12.9 | 1.50 | (1.14, 1.97) | <0.01 | 1.57 | (1.18, 2.09) | <0.01 |  |
| *Ages <65 years* |  |  |  |  |  |  |  |  |  |
| Stage I | 60 | 17.5 | Reference |  |  | Reference |  |  |  |
| Stage II | 47 | 9.1 | 1.73 | (1.07, 2.78) | 0.03 | 1.85 | (1.11, 3.08) | 0.02 | 0.56 |
| Stage III | 47 | 10.6 | 1.40 | (0.87, 2.26) | 0.17 | 1.56 | (0.92, 2.65) | 0.10 |  |
| *Ages 65-74 years* |  |  |  |  |  |  |  |  |  |
| Stage I | 62 | 24.0 | Reference |  |  | Reference |  |  |  |
| Stage II | 74 | 15.7 | 1.61 | (1.01, 2.58) | 0.05 | 1.67 | (1.01, 2.77) | 0.05 | 0.57 |
| Stage III | 56 | 12.8 | 1.98 | (1.22, 3.23) | <0.01 | 1.93 | (1.17, 3.19) | <0.01 |  |
| *Ages 75+ years* |  |  |  |  |  |  |  |  |  |
| Stage I | 47 | 18.0 | Reference |  |  | Reference |  |  |  |
| Stage II | 55 | 11.5 | 1.35 | (0.85, 2.14) | 0.20 | 1.23 | (0.77, 1.97) | 0.38 | 0.54 |
| Stage III | 49 | 14.7 | 1.17 | (0.73, 1.87) | 0.52 | 1.03 | (0.63, 1.70) | 0.89 |  |
| *Received transplant* |  |  |  |  |  |  |  |  |  |
| Stage I | 55 | 25.2 | Reference |  |  | Reference |  |  |  |
| Stage II | 47 | 9.4 | 1.57 | (0.93, 2.66) | 0.09 | 1.83 | (1.05, 3.21) | 0.03 | 0.55 |
| Stage III | 48 | 9.8 | 1.72 | (1.05, 2.82) | 0.03 | 1.96 | (1.14, 3.37) | 0.01 |  |
| *Never received transplant* |  |  |  |  |  |  |  |  |  |
| Stage I | 114 | 19.6 | Reference |  |  | Reference |  |  |  |
| Stage II | 129 | 13.7 | 1.45 | (1.06, 1.99) | 0.02 | 1.48 | (1.06, 2.07) | 0.02 | 0.54 |
| Stage III | 104 | 14.0 | 1.35 | (0.97, 1.89) | 0.08 | 1.43 | (1.01, 2.02) | 0.04 |  |
| *Diagnosed in 2016-2019* |  |  |  |  |  |  |  |  |  |
| Stage I | 85 | 22.7 | Reference |  |  | Reference |  |  | 0.56 |
| Stage II | 76 | 12.0 | 1.64 | (1.14, 2.35) | <0.01 | 1.54 | (1.05, 2.27) | 0.03 |  |
| Stage III | 73 | 12.9 | 1.71 | (1.19, 2.48) | <0.01 | 1.79 | (1.22, 2.62) | <0.01 |  |
| *Diagnosed in 2020-2022* |  |  |  |  |  |  |  |  |  |
| Stage I | 84 | 18.2 | Reference |  |  | Reference |  |  | 0.54 |
| Stage II | 100 | 11.4 | 1.38 | (0.92, 2.05) | 0.12 | 1.46 | (0.96, 2.22) | 0.08 |  |
| Stage III | 79 | 12.8 | 1.26 | (0.83, 1.91) | 0.28 | 1.32 | (0.86, 2.03) | 0.20 |  |
| *Double-hit* |  |  |  |  |  |  |  |  |  |
| Stage I | 0 | -- | -- | -- | -- | -- | -- | -- | -- |
| Stage II | 0 | -- | -- | -- | -- | -- | -- | -- |  |
| Stage III | 66 | 13.1 | -- | -- | -- | -- | -- | -- |  |
| *No double-hit* |  |  |  |  |  |  |  |  |  |
| Stage I | 169 | 20.2 | Reference |  |  | Reference |  |  | 0.56 |
| Stage II | 176 | 11.7 | 1.52 | (1.16, 1.99) | <0.01 | 1.47 | (1.11, 1.94) | <0.01 |  |
| Stage III | 86 | 12.9 | 1.65 | (1.19, 2.27) | <0.01 | 1.65 | (1.18, 2.31) | <0.01 |  |
| *Abbreviations:* CI, confidence interval; HR, hazard ratio; MASS, Mayo Additive Staging System; NR, not reached; TTNT, time to next treatment  *The multivariable model that included all patients was adjusted for age, sex, practice type and diagnosis year; and race/ethnicity was included as a stratification factor. The models by age subgroup do not include adjustment for age. The models by diagnosis year do not include adjustment for diagnosis year. | | | | | | | | | |

| **Table S8.** Prognostic performance of R2-ISS risk stratified groups overall and by age, transplantation status, year of diagnosis and double-hit status subgroups | | | | | | | | | |
| --- | --- | --- | --- | --- | --- | --- | --- | --- | --- |
| **R2-ISS risk group** | **Number of patients** | **Median TTNT (months)** | **Crude HR** | **(95% CI)** | **P** | **Adjusted HR*** | **(95% CI)** | **P** | **Harrell’s C-Index** |
| *All patients* |  |  |  |  |  |  |  |  |  |
| Stage I | 100 | 20.4 | Reference |  |  | Reference |  |  |  |
| Stage II | 126 | 15.8 | 1.05 | (0.75, 1.48) | 0.77 | 1.09 | (0.77, 1.55) | 0.62 | 0.55 |
| Stage III | 229 | 12.9 | 1.44 | (1.07, 1.93) | 0.02 | 1.51 | (1.11, 2.05) | <0.01 |  |
| Stage IV | 42 | 10.9 | 1.60 | (1.04, 2.47) | 0.03 | 1.71 | (1.09, 2.66) | 0.02 |  |
| *Ages <65 years* |  |  |  |  |  |  |  |  |  |
| Stage I | 36 | 18.3 | Reference |  |  | Reference |  |  |  |
| Stage II | 44 | 11.4 | 0.89 | (0.51, 1.54) | 0.67 | 0.91 | (0.52, 1.61) | 0.75 | 0.53 |
| Stage III | 65 | 9.1 | 1.26 | (0.77, 2.07) | 0.36 | 1.44 | (0.85, 2.47) | 0.18 |  |
| Stage IV | 9 | 11.6 | 1.54 | (0.66, 3.58) | 0.32 | 1.60 | (0.65, 3.93) | 0.30 |  |
| *Ages 65-74 years* |  |  |  |  |  |  |  |  |  |
| Stage I | 40 | 24.0 | Reference |  |  | Reference |  |  |  |
| Stage II | 50 | 22.1 | 1.32 | (0.71, 2.46) | 0.38 | 1.64 | (0.87, 3.10) | 0.13 | 0.58 |
| Stage III | 84 | 14.4 | 1.84 | (1.06, 3.21) | 0.03 | 1.92 | (1.08, 3.41) | 0.03 |  |
| Stage IV | 18 | 8.3 | 3.05 | (1.46, 6.37) | <0.01 | 3.62 | (1.70, 7.71) | <0.01 |  |
| *Ages 75+ years* |  |  |  |  |  |  |  |  |  |
| Stage I | 24 | 18.0 | Reference |  |  | Reference |  |  |  |
| Stage II | 32 | 11.0 | 1.03 | (0.55, 1.92) | 0.93 | 0.90 | (0.47, 1.73) | 0.75 | 0.53 |
| Stage III | 80 | 13.7 | 1.28 | (0.76, 2.14) | 0.36 | 1.12 | (0.65, 1.93) | 0.69 |  |
| Stage IV | 15 | 14.7 | 0.91 | (0.43, 1.92) | 0.81 | 0.69 | (0.30, 1.59) | 0.38 |  |
| *Received transplant* |  |  |  |  |  |  |  |  |  |
| Stage I | 32 | 25.2 | Reference |  |  | Reference |  |  |  |
| Stage II | 43 | 22.0 | 0.88 | (0.47, 1.65) | 0.69 | 1.08 | (0.56, 2.10) | 0.81 | 0.57 |
| Stage III | 61 | 9.4 | 1.52 | (0.87, 2.65) | 0.14 | 1.85 | (1.01, 3.40) | 0.05 |  |
| Stage IV | 14 | 8.3 | 2.39 | (1.14, 5.03) | 0.02 | 2.55 | (1.14, 5.74) | 0.02 |  |
| *Never received transplant* |  |  |  |  |  |  |  |  |  |
| Stage I | 68 | 20.2 | Reference |  |  | Reference |  |  |  |
| Stage II | 83 | 12.7 | 1.17 | (0.78, 1.76) | 0.46 | 1.18 | (0.78, 1.80) | 0.43 | 0.54 |
| Stage III | 168 | 14.0 | 1.39 | (0.98, 1.98) | 0.07 | 1.40 | (0.97, 2.03) | 0.07 |  |
| Stage IV | 28 | 14.3 | 1.24 | (0.72, 2.13) | 0.44 | 1.30 | (0.74, 2.30) | 0.37 |  |
| *Diagnosed in 2016-2019* |  |  |  |  |  |  |  |  |  |
| Stage I | 51 | 20.2 | Reference |  |  | Reference |  |  | 0.55 |
| Stage II | 52 | 19.7 | 0.86 | (0.54, 1.38) | 0.54 | 0.94 | (0.58, 1.52) | 0.80 |  |
| Stage III | 110 | 12.9 | 1.43 | (0.98, 2.10) | 0.07 | 1.50 | (1.01, 2.23) | 0.04 |  |
| Stage IV | 21 | 10.9 | 1.56 | (0.88, 2.77) | 0.13 | 1.84 | (1.01, 3.35) | 0.05 |  |
| *Diagnosed in 2020-2022* |  |  |  |  |  |  |  |  |  |
| Stage I | 49 | 20.4 | Reference |  |  | Reference |  |  | 0.54 |
| Stage II | 74 | 11.4 | 1.31 | (0.78, 2.19) | 0.31 | 1.32 | (0.78, 2.26) | 0.30 |  |
| Stage III | 119 | 12.8 | 1.46 | (0.91, 2.33) | 0.12 | 1.51 | (0.92, 2.45) | 0.10 |  |
| Stage IV | 21 | 10.1 | 1.68 | (0.86, 3.26) | 0.13 | 1.65 | (0.83, 3.25) | 0.15 |  |
| *Double-hit* |  |  |  |  |  |  |  |  |  |
| Stage I | 0 | -- | -- |  |  | -- |  |  | 0.52 |
| Stage II | 1 | NR | -- |  |  | -- |  |  |  |
| Stage III | 37 | 16.8 | Reference |  |  | Reference |  |  |  |
| Stage IV | 28 | 10.9 | 1.19 | (0.66, 2.15) | 0.56 | 1.44 | (0.75, 2.76) | 0.27 |  |
| *No double-hit* |  |  |  |  |  |  |  |  |  |
| Stage I | 100 | 20.4 | Reference |  |  | Reference |  |  | 0.55 |
| Stage II | 125 | 15.8 | 1.07 | (0.76, 1.50) | 0.72 | 1.09 | (0.77, 1.55) | 0.62 |  |
| Stage III | 192 | 12.8 | 1.49 | (1.09, 2.02) | 0.01 | 1.51 | (1.10, 2.07) | 0.01 |  |
| Stage IV | 14 | 10.6 | 2.00 | (1.07, 3.72) | 0.03 | 1.95 | (1.03, 3.70) | 0.04 |  |
| *Abbreviations:* CI, confidence interval; HR, hazard ratio; NR, not reached; R2-ISS, Second Revision of the International Staging System; TTNT, time to next treatment  *The multivariable model that included all patients was adjusted for age, sex, practice type and diagnosis year; and race/ethnicity was included as a stratification factor. The models by age subgroup do not include adjustment for age. The models by diagnosis year do not include adjustment for diagnosis year. | | | | | | | | | |

**Table S9 that explores association of treatment utilization with stages.**

|  | **All NDMM Patients (N=497)** | | **Stage I** | | **Stage II** | | **Stage III** | | **Stage IV** | | **p-value** |
| --- | --- | --- | --- | --- | --- | --- | --- | --- | --- | --- | --- |
|  | n | (%) | n | (%) | n | (%) | n | (%) | n | (%) |  |
| **R-ISS** | 497 | (100.0) | 122 | (24.5) | 311 | (62.6) | 64 | (12.9) | NA | |  |
| **First-line treatment type** |  |  |  | |  | |  | |  | | 0.41 |
| Doublet | 69 | (13.9) | 23 | (18.9) | 39 | (12.5) | 7 | (10.9) |  |  |  |
| Triplet | 336 | (67.6) | 75 | (61.5) | 213 | (68.5) | 48 | (75.0) |  |  |  |
| Quad | 53 | (10.7) | 16 | (13.1) | 32 | (10.3) | 5 | (7.8) |  |  |  |
| Other* | 39 | (7.8) | 8 | (6.6) | 27 | (8.7) | 4 | (6.2) |  |  |  |
| **Autologous stem cell transplantation** |  |  |  |  |  |  |  |  |  |  | 0.12 |
| Up to 1-year post-initiation | 150 | (30.2) | 41 | (33.6) | 93 | (29.9) | 16 | (25.0) |  |  |  |
| >1 year post-initiation | 17 | (3.4) | 8 | (6.6) | 8 | (2.6) | 1 | (1.6) |  |  |  |
| Not transplanted | 330 | (66.4) | 73 | (59.8) | 210 | (67.5) | 47 | (73.4) |  |  |  |
|  |  |  |  |  |  |  |  |  |  |  |  |
| **MASS** | 497 | (100.0) | 169 | (34.0) | 176 | (35.4) | 152 | (30.6) | NA | |  |
| **First-line treatment type** |  |  |  |  |  |  |  |  |  |  |  |
| Doublet | 69 | (13.9) | 24 | (14.2) | 24 | (13.6) | 21 | (13.8) |  |  | 0.94 |
| Triplet | 336 | (67.6) | 111 | (65.7) | 117 | (66.5) | 108 | (71.1) |  |  |  |
| Quad | 53 | (10.7) | 20 | (11.8) | 20 | (11.4) | 13 | (8.6) |  |  |  |
| Other* | 39 | (7.8) | 14 | (8.3) | 15 | (8.5) | 10 | (6.6) |  |  |  |
| **Autologous stem cell transplantation** |  |  |  |  |  |  |  |  |  |  |  |
| Up to 1-year post-initiation | 150 | (30.2) | 55 | (32.5) | 47 | (26.7) | 48 | (31.6) |  |  | 0.14 |
| >1 year post-initiation | 17 | (3.4) | 10 | (5.9) | 4 | (2.3) | 3 | (2.0) |  |  |  |
| Not transplanted | 330 | (66.4) | 104 | (61.5) | 125 | (71.0) | 101 | (66.4) |  |  |  |
|  |  |  |  |  |  |  |  |  |  |  |  |
| **R2-ISS** | 497 | (100.0) | 100 | (20.1) | 126 | (25.4) | 229 | (46.1) | 42 | (8.5) |  |
| **First-line treatment type** |  |  |  |  |  |  |  |  |  |  | 0.90 |
| Doublet | 69 | (13.9) | 15 | (15.0) | 20 | (15.9) | 30 | (13.1) | 4 | (9.5) |  |
| Triplet | 336 | (67.6) | 66 | (66.0) | 79 | (62.7) | 160 | (69.9) | 31 | (73.8) |  |
| Quad | 53 | (10.7) | 12 | (12.0) | 16 | (12.7) | 20 | (8.7) | 5 | (11.9) |  |
| Other* | 39 | (7.8) | 7 | (7.0) | 11 | (8.7) | 19 | (8.3) | 2 | (4.8) |  |
| **Autologous stem cell transplantation** |  |  |  |  |  |  |  |  |  |  |  |
| Up to 1-year post-initiation | 150 | (30.2) | 32 | (32.0) | 43 | (34.1) | 61 | (26.6) | 14 | (33.3) | 0.19 |
| >1 year post-initiation | 17 | (3.4) | 7 | (7.0) | 4 | (3.2) | 6 | (2.6) | 0 | (0.0) |  |
| Not transplanted | 330 | (66.4) | 61 | (61.0) | 79 | (62.7) | 162 | (70.7) | 28 | (66.7) |  |

*Patients receiving either monotherapy or a clinical study drug were categorized as “Other”

| **Table S10.** Overall survival in high-risk cytogenetic abnormality risk-stratified groups overall | | | | | | | | | |
| --- | --- | --- | --- | --- | --- | --- | --- | --- | --- |
| **HRCA risk group** | **Number of patients** | **Median OS (months)** | **Crude HR** | **(95% CI)** | **P** | **Adjusted HR*** | **(95% CI)** | **P** | **Harrell’s C-Index** |
| *All patients* |  |  |  |  |  |  |  |  |  |
| HRCA 0 | 284 | 69.3 | Reference |  |  | Reference |  |  |  |
| HRCA 1 | 147 | 56.2 | 1.39 | (0.95, 2.05) | 0.09 | 1.32 | (0.90, 1.95) | 0.16 | 0.55 |
| HRCA 2+ | 66 | 41.4 | 1.84 | (1.18, 2.87) | 0.01 | 1.81 | (1.14, 2.87) | 0.01 |  |
| *Abbreviations:* CI, confidence interval; HR, hazard ratio; HRCA, high-risk cytogenetic abnormality; NR, not reached; OS, overall survival  *The multivariable model that included all patients was adjusted for race/ethnicity, sex, practice type and diagnosis year; and age was included as a stratification factor. | | | | | | | | | |

| **Table S11.** Time to next treatment in high-risk cytogenetic abnormality risk-stratified groups overall | | | | | | | | | |
| --- | --- | --- | --- | --- | --- | --- | --- | --- | --- |
| **HRCA risk group** | **Number of patients** | **Median TTNT (months)** | **Crude HR** | **(95% CI)** | **P** | **Adjusted HR*** | **(95% CI)** | **P** | **Harrell’s C-Index** |
| *All patients* |  |  |  |  |  |  |  |  |  |
| HRCA 0 | 284 | 17.3 | Reference |  |  | Reference |  |  |  |
| HRCA 1 | 147 | 12.8 | 1.39 | (1.09, 1.77) | 0.01 | 1.36 | (1.06, 1.74) | 0.02 | 0.53 |
| HRCA 2+ | 66 | 13.1 | 1.15 | (0.83, 1.59) | 0.41 | 1.23 | (0.88, 1.72) | 0.23 |  |
| *Abbreviations:* CI, confidence interval; HR, hazard ratio; HRCA, high-risk cytogenetic abnormality; NR, not reached; TTNT, time to next treatment  *The multivariable model that included all patients was adjusted for age, sex, practice type and diagnosis year; and race/ethnicity was included as a stratification factor. | | | | | | | | | |
